# Pleasantness emotions and neural activity induced by the multimodal crispiness of monaka ice cream

**DOI:** 10.3389/fnut.2025.1681999

**Published:** 2025-11-13

**Authors:** Takayuki Yamamoto, Ryo Tanaka, Takanori Kochiyama, Yukiko Nota, Hitomi Tsuchiya, Masato Kawamoto, Wataru Sato

**Affiliations:** 1 R&D Institute, Morinaga & Co., Ltd., Yokohama, Japan; 2 Brain Activity Imaging Center, ATR-Promotions, Kyoto, Japan; 3 Psychological Process Research Team, Guardian Robot Project, RIKEN, Kyoto, Japan

**Keywords:** monaka ice cream, functional MRI, texture perception, moisture content, hedonic valuation, auditory cortex, ventromedial prefrontal cortex

## Abstract

Monaka ice cream is a frozen confection consisting of ice cream sandwiched between two wafers. Previous psychological research has reported that monaka ice cream with a relatively low moisture content delivers crispiness and enhances positive emotions during consumption. However, the neural basis of these subjective impressions remains unclear. Therefore, this study aimed to investigate the neural mechanisms through which crispy monaka ice cream evokes pleasurable emotions. Ten healthy adults participated in the study. The participants first consumed two types of monaka ice cream (low moisture content, approximately 7%; and high moisture content, approximately 12%) in random order. The consumption method involved breaking the ice cream in half by hand and eating three bites per video samples. Subsequently, we performed functional magnetic resonance imaging (fMRI) while the participants watched multimodal (visual + auditory) videos of the low- and high-moisture-content monaka ice cream, recalling their eating experiences. During fMRI image acquisition, the participants rated their preference levels on a four-point scale. Preference ratings were higher for low-moisture-content monaka ice cream than for high-moisture-content cream. Lower moisture contents elicited stronger activity in the bilateral auditory cortex and bilateral ventromedial prefrontal cortex (vmPFC) compared with the high moisture content condition. Additionally, preference ratings were positively correlated with vmPFC activity. These findings suggest that the multimodal processing of crispy monaka ice cream involves activity in the auditory cortex and vmPFC, the latter of which contributes to the generation of subjective pleasurable emotions.

## 1 Introduction

Monaka ice cream is a traditional Japanese frozen confectionery consisting of ice cream sandwiched between thin wafers. These wafers are called “monaka,” originally used in Japanese sweets to encase sweetened bean paste. Monaka dates back to the Heian period (8th to 9th centuries CE), and has evolved into various forms, becoming popular among the general public during the Edo period (17th century). Monaka ice cream emerged through the fusion of monaka and Western ice cream. The primary ingredients of monaka wafers include wheat flour, modified starch, and glutinous rice flour, resulting in a light and crispy texture when baked. The ice cream is encased in the monaka wafer, which is shaped with the fine holes in the wafers, minimizing the contact surface area. This structural feature inhibits heat transfer from the hands to the ice cream. As a result, monaka ice cream can be consumed easily while keeping the hands clean. During manufacturing, the batter is poured into molds and baked to create lightweight edible monaka containers. Subsequently, the soft ice cream is molded and filled into monaka containers, which are then frozen until fully solidified. When consumed, the ice cream remains firmly encased in the monaka, providing a unique combination of textures: firm ice cream, light, and crispy wafers. Monaka ice cream, with a light texture similar to that of cone ice cream and wafer ice cream, along with the variety of available flavors, is enjoyed by people of all ages. Recently, it has gained attention not only in Japan but also overseas, where its unique taste and texture are highly appreciated.

Previous psychological studies have reported that adjusting the moisture content of the wafer in monaka ice cream changes the crispy texture changes, which is accompanied by changes in emotion during consumption (1). For example, a previous study has demonstrated that consuming monaka ice cream with a lower moisture content in the wafer results in more positive emotions (2). Several studies on other foods have shown that crispiness is influenced by multisensory inputs, including visual, auditory, and tactile information. For example, one study investigated how the sound produced when biting potato chips affects consumers' perceptions of crispiness and freshness (3). The results demonstrated that increases in the loudness and high-frequency components of the biting sound directly enhanced the perceived crispiness and freshness; specifically, the amplification of high-frequency sounds and overall volume tended to make potato chips seem crisper and fresher. Another study examined the effects of visual and auditory information on the perception of biscuit crispiness (4). By masking both sensory modalities, this study assessed the consequences of perceived crispiness and masticatory physiology. The results indicated that masking both visual and auditory cues reduced crispiness ratings, with auditory masking having a particularly pronounced effect, whereas the impact of visual masking was more limited. These findings highlight the crucial role of auditory information in the sensory evaluation of foods. Collectively, these findings suggest that low-moisture monaka ice cream delivers a crispier texture and enhances positive emotions during consumption specifically because of its sound.

However, the neural mechanisms underlying the pleasant subjective emotions evoked by the crispiness of monaka ice cream remain unclear. Previous studies using functional magnetic resonance imaging (fMRI) have indicated that the limbic regions, primarily the ventromedial prefrontal cortex (vmPFC), are responsive to various gustatory information, playing a role in food recognition and hedonic evaluation. The vmPFC serves as a multisensory hub that receives inputs from sensory modalities such as gustation, olfaction, audition, vision, and somatic sensation and primarily projects to the ventral striatum (5). Early fMRI studies have consistently demonstrated that activation of the vmPFC correlates with subjective reports of pleasure in response to various types of rewards, including primary, secondary, and abstract (6–10). The vmPFC is believed to integrate signals from different sensory modalities and represents them on a common scale reflecting the attractiveness or value of rewards, thereby enabling comparison and evaluation (11). Based on these findings, we hypothesized that the vmPFC integrates multimodal information and produces pleasant subjective emotions during the multimodal processing of crispy monaka ice cream.

To test this hypothesis, we measured fMRI while participants observed multimodal (visual + auditory) videos of the two types of monaka ice cream—one with low (7%) and one with high (12%) moisture content—after they consumed them and experienced the differences. Based on previous psychological research (2), we expected that monaka ice cream with lower moisture content would provide a crispier texture and enhance positive emotions. During the fMRI measurement, the participants rated their preference on a four-point scale. This approach enabled the investigation of both neural and subjective responses to variations in wafer moisture content. We predicted that pleasurable emotions and neural activity induced by the multimodal crispiness of monaka ice cream would be concerned.

## 2 Method

### 2.1 Study framework

This study was conducted in accordance with the principles of the Declaration of Helsinki (amended October 2013). It also complied with the Ethical Guidelines for Medical and Health Research Involving Human Subjects (notified by the Ministry of Education, Culture, Sports, Science and Technology and the Ministry of Health, Labour and Welfare; issued December 22, 2014, partially revised February 28, 2017) as well as its related guidance documents (issued on February 9, 2015, partially revised March 8, 2017).

Approval for this study was obtained from the Institutional Review Board of Shiba Palace Clinic (IRB number: 16000008) and the fMRI Safety Review Board of ATR-Promotions, Inc. Prior to participation, all subjects were fully informed of the study objectives, content, and procedures, and written informed consent was obtained.

The recruitment and management of participants, as well as the establishment of the study's operational framework, were conducted by Agekke Co., Ltd., and fMRI measurements were performed at ATR-Promotions, Inc. The study was conducted from January to March 2023 and registered in a public database (UMIN Clinical Trials Registry) under UMIN ID: UMIN000053549.

### 2.2 Participants

**Figure 1:**
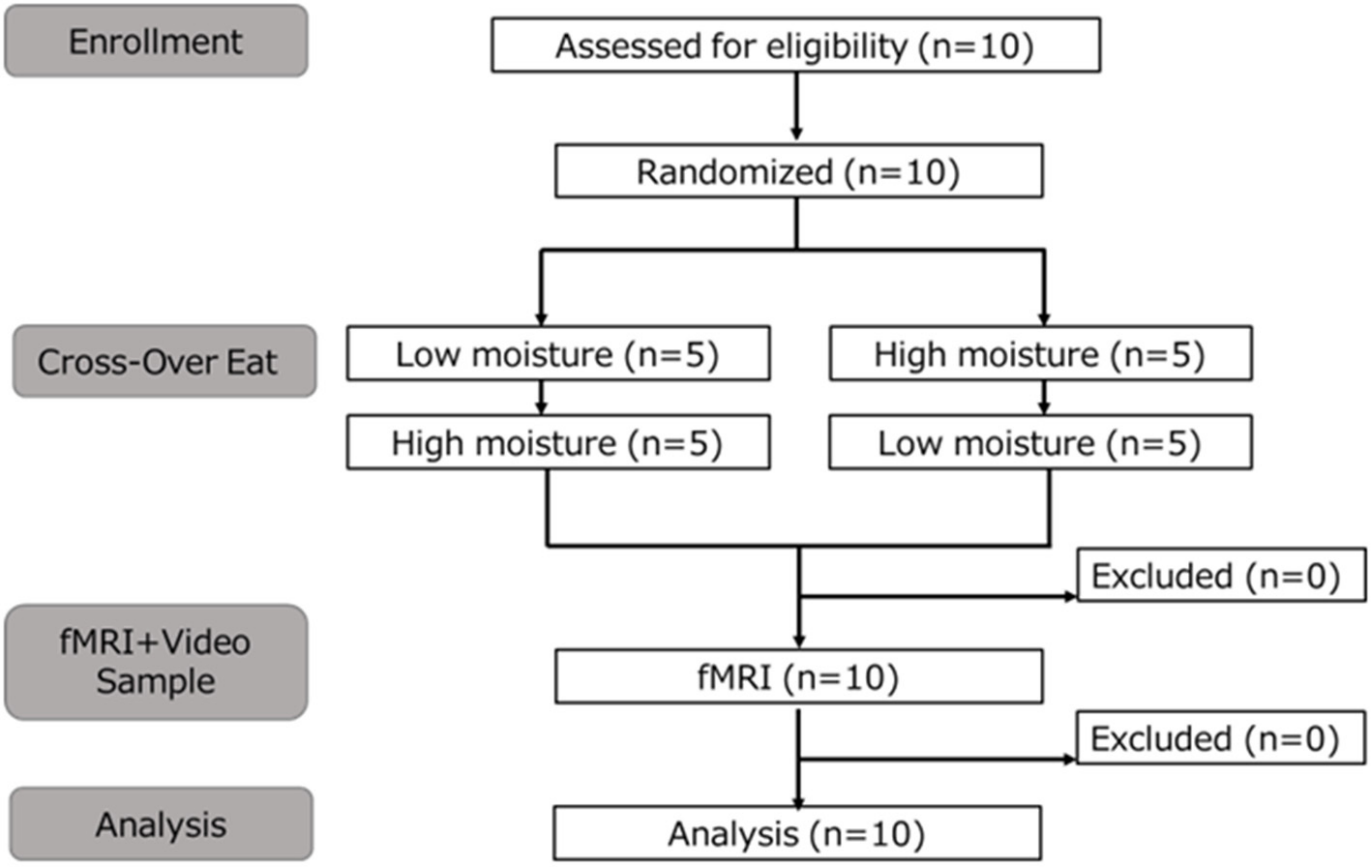
Flow chart of the study design from recruitment to analysis.

This study tested 10 healthy adults (six males, four females; mean ± standard deviation, 27.7 ± 6.0 years). The sample size was determined based on the work of de Araujo and Rolls (12), who investigated brain responses to food texture using fMRI. Accordingly, the number of participants was set at 10, selected within the feasible range for this study. All participants were right-handed and had previously been subjected to fMRI investigations. The flow of participant selection and analysis is shown in Figure 1. The inclusion and exclusion criteria were as follows.

#### 2.2.1 Inclusion criteria

(1) Previous experience of consuming and enjoying monaka ice cream, the test food

(2) Individuals who have previously undergone MRI investigation

(3) Right-handed individuals

(4) Healthy individuals without chronic physical illness

(5) Individuals who received sufficient explanation regarding the purpose and content of the study demonstrated adequate comprehension and capacity for consent and voluntarily provided written informed consent.

(6) Individuals could attend scheduled measurement days and undergo assessments.

#### 2.2.2 Exclusion criteria

(1) Individuals currently undergoing pharmacological treatment for any illness

(2) Individuals with a medical history that may affect study participation as determined by investigators

(3) Individuals with alcohol dependence or other psychiatric disorders

(4) Individuals with a habitual intake of medications for disease treatment within the past month (excluding occasional use for headache, menstrual pain, or common cold)

(5) Individuals with food allergies (including history thereof)

(6) Individuals with a current smoking habit

(7) Female participants who are pregnant, lactating, possible pregnant, or planning pregnancy during the study period

(8) Individuals with a history of brain surgery or brain diseases

(9) Individuals with claustrophobia

(10) Individuals with tattoos

(11) Individuals with orthodontic devices, implants, or internal metals such as plates or screws for fracture repair

(12) Individuals who have received inpatient treatment within the past 6 months

(13) Individuals currently participating in another clinical trial or within 3 months of participation in another clinical trial.

### 2.3 Test foods

**Figure 2:**
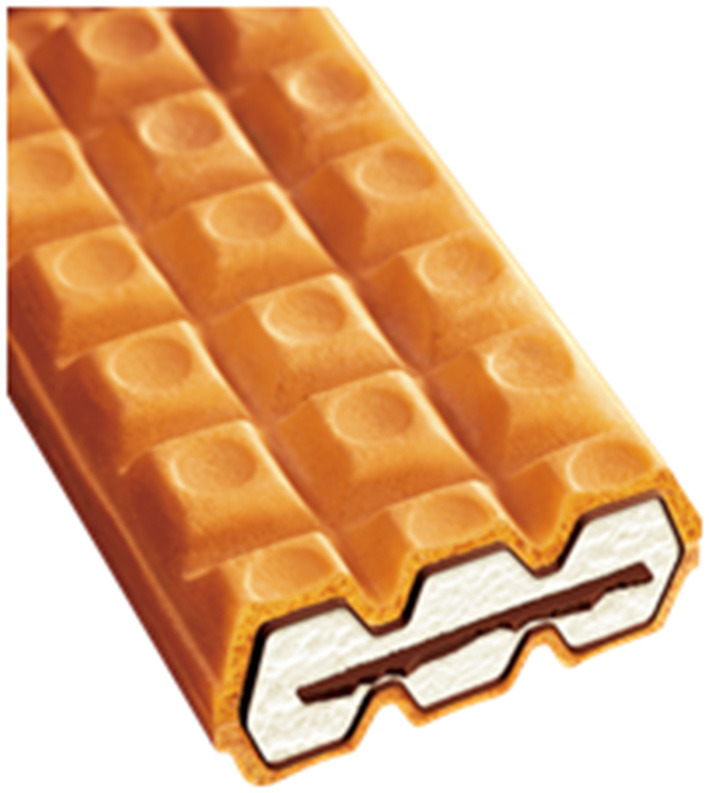
Picture of the monaka ice cream used in this study. The only difference between samples was the moisture content of the monaka portion. Each participant took three bites of the exposed portion of either sample before fMRI acquisition.

Two types of monaka ice cream (both manufactured by Morinaga & Co., Ltd.) were used in this study: one with a high wafer moisture content (12%) and one with a low wafer moisture content (7%). The compositions of the test foods were identical, except for the moisture content of the wafer. An image and the nutritional components of the test foods are shown in Figure 2 and Table 1.

**Table 1:** Ingredients and nutritional components of the test foods.

Kind	Test food
Form	Complex frozen snacks (Monaka ice cream)
Raw materials	Chocolate coating (made in Japan), sugar, monaka (contains wheat and eggs), dairy products, vegetable oil, starch syrup, dextrin, salt/modified starch, emulsifier (soybean-derived), stabilizer (thickening polysaccharide), flavoring, annatto color, carotene color
Allergens	Eggs, milk, wheat
**Nutritional Information (per 100g)**	
Energy (kcal)	103
Protein (g)	1.2
Fat (g)	6.1
Carbohydrates (g)	11.4
Salt equivalent Na (g)	0.04

### 2.4 Video samples

Six employees of Morinaga & Co., Ltd. (three females and three males) participated in the video recording. Each video participant performed two actions: breaking the monaka ice cream in half by hand and eating the broken piece. The former was filmed with a focus on the hands, whereas the latter was filmed with a focus on the mouth. These footage segments were then edited by trimming 8 s of the breaking scene and 8 s of the eating scene and concatenating them to create 16-s videos (Supplementary Movies S1). In total, 12 videos were prepared (2 types of monaka ice cream × 6 participants). Additionally, high-performance microphones were used during the recording to capture both breaking and eating sounds. To ensure that all the participants could clearly perceive the auditory stimuli despite the scanner noise, a dummy scan was conducted at the beginning of the functional runs. During this period, the experimenter adjusted the volume of the auditory stimuli for each participant until the participants confirmed that the sounds were audible and comfortable. This procedure was implemented to ensure the validity of auditory stimulus presentation during fMRI acquisition.

### 2.5 Video presentation apparatus

The experimental events were controlled using Presentation Software (version 24.0; Neurobehavioral Systems, Inc., Berkley, CA, USA), and the visual stimuli were projected from a liquid crystal projector (WUX6000; Canon Inc., Tokyo Japan) onto a mirror positioned in a scanner in front of the participant. Auditory stimuli were presented using an MRI-compatible headphone system (Kobatel Corporation, Yokohama, Japan).

### 2.6 Study design

After obtaining informed consent, the participants consumed high- and low-moisture monaka ice creams in randomized order. Consumption followed the procedure shown in the video samples: first, the monaka ice cream was broken half by hand, and then the exposed portion was taken in three bites. Subsequently, the participants moved to the fMRI room, changed clothes, wore headphones, and entered the scanner while holding a response device in their right hand for preference ratings. The brain activity was also recorded.

During fMRI scanning, participants were shown prerecorded video samples inside the scanner, and sounds of breaking and eating were played through headphones. The video presentations consisted of two sessions. In each session, participants viewed a 16-s breaking/eating video, followed by a 2-s period for rating preference using the button device before a 16-s rest. This sequence was repeated for all 12 video types. The videos of high- and low-moisture monaka ice cream were alternately presented: in session one, the order was “low moisture → high moisture → low moisture → ...”, and in session two, the order was reversed. The sex of each individual in the video was randomized across the 12 samples.

While viewing each video, the participants were instructed to recall the sensory experience of eating the corresponding type of monaka ice cream prior to scanning. Comfort was rated on a four-point scale (“strongly agree,” “somewhat agree,” “somewhat disagree,” “strongly disagree”) a total of 24 times. Finally, structural MRI images were acquired.

### 2.7 MRI acquisition

Imaging was performed using a 3-T scanning system (MAGNETOM Prisma, Siemens Medical Solutions, Malvern, PA, USA) at the ATR Brain Activity Imaging Center using a 64-channel head coil. Small elastic pads were placed on both sides of the head to stabilize its position. The functional images consisted of 60 consecutive axial slices, with a 0.5 mm gap and covering the whole brain. A T2^*^-weighted multiband gradient-echo echo-planar imaging (EPI) sequence was used with the following parameters: repetition time (TR) = 2000; echo time (TE) = 30 ms; flip angle = 80°; multiband acceleration factor = 3; partial Fourier = 6/8; matrix size = 100 × 100; voxel size = 2 × 2 × 2 mm. After acquiring the functional images, a T1-weighted high-resolution anatomical image was obtained using a magnetization-prepared rapid-acquisition gradient-echo (MPRAGE) sequence (TR = 2250 ms; TE = 3.06 ms; flip angle = 9°; GRAPPA acceleration factor = 2; 208 sagittal slices; field of view = 256 × 256 mm; voxel size = 1 × 1 × 1 mm).

### 2.8 Image analysis

Image analyses were performed using the Statistical Parametric Mapping package (SPM12; revision 7771; http://www.fil.ion.ucl.ac.uk/spm), implemented in MATLAB R2022b (MathWorks).

For pre-processing, all functional images were corrected for slice timing. Next, the functional images were realigned to the first image of each run, and then to the mean image of the entire run as a reference to correct for head motion. The realignment parameters revealed only a small motion correction (< 2 mm). The anatomical images were then coregistered with the mean functional image. Subsequently, all anatomical and functional images were spatially normalized to the Montreal Neurological Institute (MNI) space using an anatomical image-based unified segmentation-spatial normalization approach. Finally, the spatially normalized functional images were resampled to a voxel size of 2 × 2 × 2 mm and smoothed with an 8 mm full width at half-maximum isotropic Gaussian kernel to compensate for anatomical variability among participants.

Random-effects analyses were performed to identify significantly activated voxels at the population level (13). First, we performed a single-subject analysis using a general linear model (GLM). The task conditions and responses were embedded in a series of boxcar and delta functions, respectively. The task-related regressor was modeled using a convolution with a canonical hemodynamic response function. We used a high-pass filter composed of a discrete cosine basis function with a cutoff period of 128 s to eliminate artifactual low-frequency trends. To reduce motion-related noise and other confounding noise, such as physiological noise, additional nuisance regression was conducted using the PhysIO Toolbox (version R2020a-v7.3.0 (14), which is part of the Translational Algorithms for Psychiatry-Advancing Science software collection (https://github.com/ComputationalPsychiatry). The nuisance regressors included six head motion parameters generated by the realignment step and six white matter (WM) and six cerebrospinal fluid (CSF)-related time courses. WM and CSF signals across voxels in the associated mask were extracted for each participant, and the average and first five principal components of the WM and CSF signals were used as nuisance regressors based on the CompCor method (15). Serial autocorrelation, assuming a first-order autoregressive model, was estimated from the pooled active voxels using a restricted maximum likelihood procedure and was used to whiten the data and design matrix. The contrast images of each task condition vs. baseline from the first-level single-subject analysis were entered into a full factorial model for the second-level random effects analysis.

To identify the brain regions associated with individual preference ratings, we conducted a parametric modulation analysis (16), wherein another GLM was constructed for each participant. All task blocks (i.e., stimulus presentations) were modeled as task regressors. Individual preference rating scores were used as the first-order (linear) parametric modulators for each task block. The parametric modulator was entered as a separate regressor in the design matrix and was orthogonalized with respect to the main task regressor to ensure that the modulation effect was independent of the task-evoked response. The task regressor and parametric modulator are convolved with the canonical hemodynamic response function. This approach allows the model to capture block-by-block variations in BOLD responses as a function of subjective ratings, thereby identifying brain regions in which activity covaries with preference intensity. Other nuisance regressors (realignment parameters and CompCor regressors), high-pass filters, and serial autocorrelations were applied using the aforementioned settings. The contrast images of the task regressor and parametric modulator from the single-subject analysis were entered into a full factorial model for second-level random effects analysis.

Initially, the contrast between high- and low-wafer-moisture content conditions was examined at the second level. Next, a positive linear relationship between brain activity and preference ratings was tested using parametric modulation analysis. We identified significant clusters at *p* < 0.05, family wise error (FWE)-corrected for multiple comparisons over the whole brain, with a cluster-forming threshold of *p* < 0.001, uncorrected (17, 18). Although stringent correction increases the risk of Type II errors in small samples, we deliberately adopted a conservative approach, reporting only those effects that were sufficiently robust to withstand rigorous control for false positives, even at the cost of reduced sensitivity. The brain structures were anatomically labeled and identified according to Brodmann's area using the Automated Anatomical Labelling atlas (19, 20) and Brodmann maps (Brodmann.nii) provided by MRIcron software (https://people.cas.sc.edu/rorden/mricron/index.html). The area of activation is superimposed on the mean normalized structural images of the participants to illustrate the anatomical regions in the figures.

## 3 Results

### 3.1 The preference rating scores

**Figure 3:**
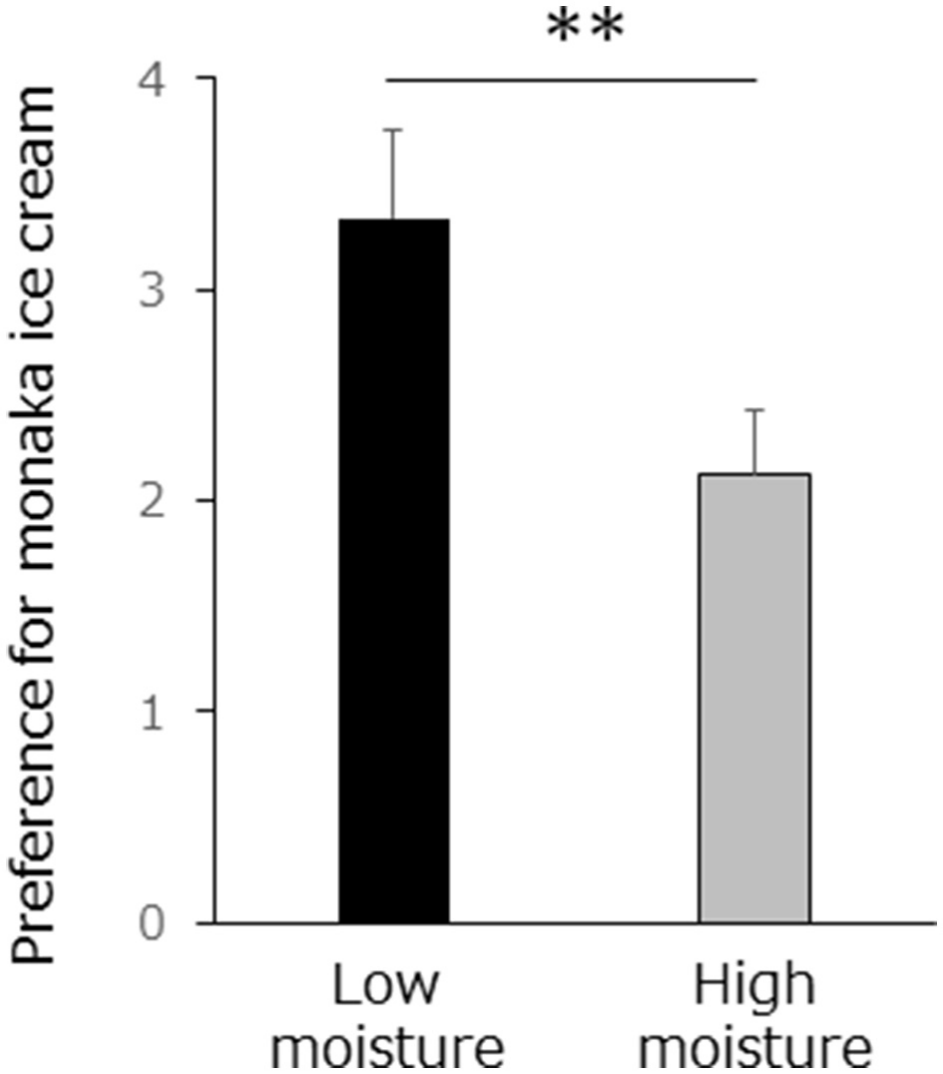
Preference was rated on a four–point scale (4, “strongly agree,” 3, “somewhat agree,” 2, “somewhat disagree,” 1, “strongly disagree”) preference with high or low moisture monaka was evaluated six times per session, for a total of four sessions. Values are means ± SD for 10 samples. **p < 0.01.

The evaluation of the low-moisture monaka ice cream was significantly higher than that of the high-moisture monaka ice cream (*t*-test, *p* < 0.001; Figure 3). In addition, there was no significant difference in reaction times between the high-moisture and low-moisture conditions (mean ± standard deviation, 1.23 ± 0.25 and 1.30 ± 0.30 s, respectively) during the interval from the presentation of the rating screen to the button press (*t*-test, *p* = 0.36).

### 3.2 Regional brain activity

**Figure 4:**
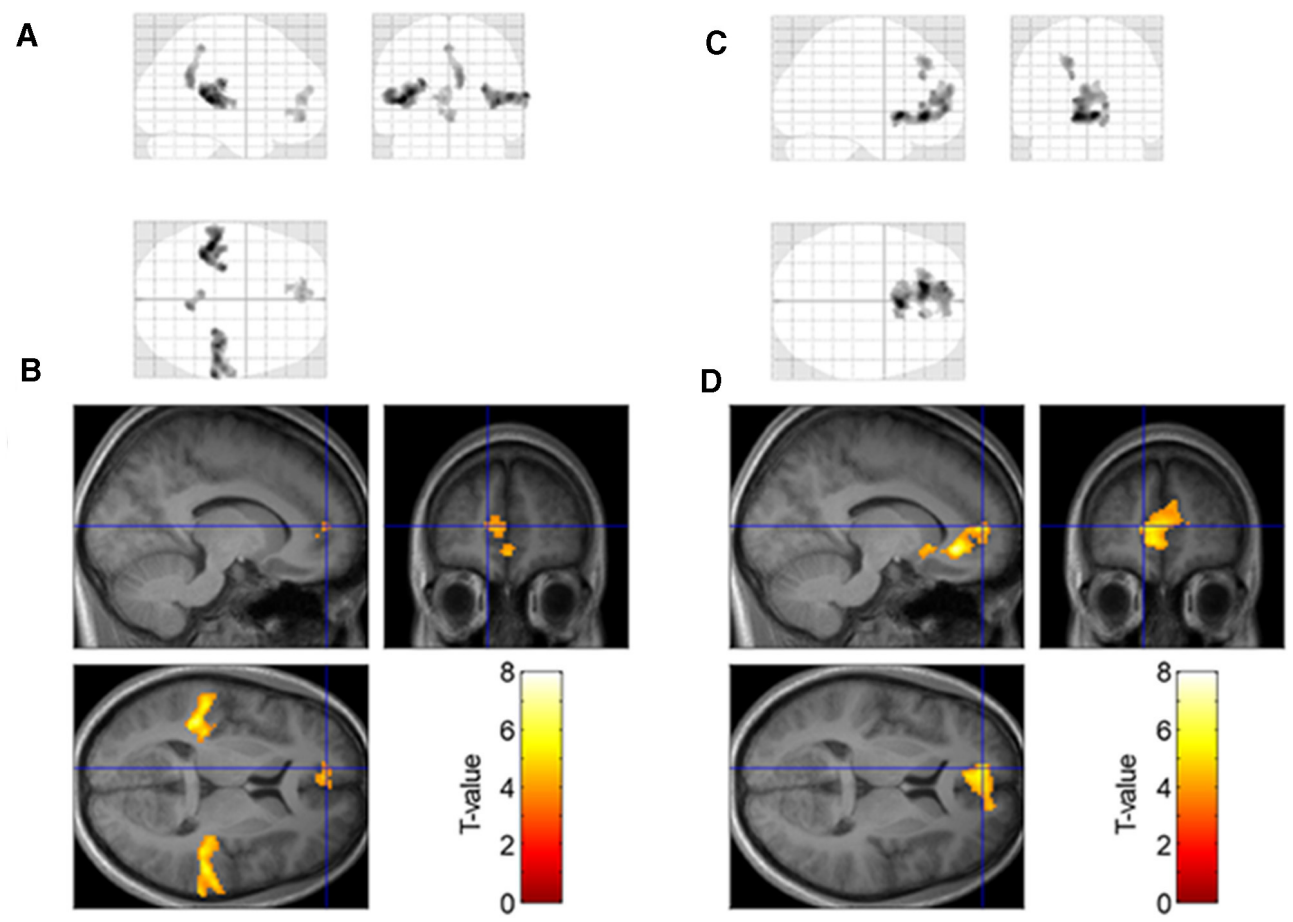
Brain regions significant activation in response to low moisture vs. high moisture samples **(A, B)** and significant relationship with preference rating scores **(C, D)** at *p* < 0.05 cluster–level FWE–corrected for multiple comparisons. The red–yellow color scale indicates the *T*–values. The blue cross indicates the location of the medial prefrontal cortex (mPFC)(x = −12, y = 50, z = 8).

We tested the contrast between low- and high-moisture monaka ice cream and found significant activation of the bilateral auditory cortex (superior temporal gyrus), precuneus, and medial prefrontal cortex (medial orbitofrontal cortex, pregenual anterior cingulate cortex, and medial superior frontal gyrus) (Figures 4A, B, Table 2).

**Table 2:** Brain regions that exhibited significant activation and parametric modulation.

**Contrast**	**Region**	**BA**	**Coordinates**			***T_18_*-value**	**Cluster Size**
			***x***	***y***	***z***		**(voxels)**
Low moisture vs. High moisture	L. Superior temporal gyrus	41	−44	−34	6	6.63	693
	R. Superior temporal gyrus	48	42	−28	6	5.92	563
	R. Precuneus	23	8	−54	28	5.00	184
	Medial orbitofrontal cortex	11	0	48	−10	4.51	248
	L. Pregenual anterior cingulate cortex	32	−6	48	8	4.44	
	L. Medial superior frontal gyrus	10	−12	50	8	4.30	
Parametrc modulation by preference score	L. Subgenual anterior cingulate cortex	11	−10	34	−6	7.26	1,628
	L. Pregenual anterior cingulate cortex	10	−8	52	0	6.00	
	L. Medial superior frontal gyrus	10	−12	50	8	5.65	

BA, Brodmann's area.

Parametric modulation analysis showed that the brain regions in which the signal was positively correlated with preference rating scores were located in the medial prefrontal cortex (Figures 4C, D, Table 2). This region spatially overlapped with those identified by contrasts between the low- and high-moisture monaka ice creams.

## 4 Discussion

In this study, we investigated the relationship between emotions and brain activity elicited by monaka ice cream with different moisture contents. Immediately after consuming each type, the participants viewed a video depicting the same eating procedure. The participants were not informed about the existence of the two types of videos (low- and high-moisture content), nor were they given any information about the videos prior to viewing. The preference ratings for low-moisture monaka ice cream were significantly higher than those for high-moisture monaka ice cream (Figure 3). Generally, foods with a crispy texture elicit positive emotions, although some individuals may prefer soft or soggy textures. Nonetheless, the present study findings suggest that “pleasantness” was elicited by crispy audiovisual cues regardless of subjective preference.

More importantly, the fMRI results revealed that activity in the auditory cortex was greater when participants watched low-moisture monaka ice cream videos than when they watched high-moisture monaka ice cream videos. Sound is an important feature of pleasurable eating experiences. Research on potato chips has shown that increasing the loudness and high-frequency components of the biting sound significantly enhances the perceived crispiness and freshness, highlighting the importance of auditory information in sensory evaluation (3). Similarly, studies on biscuits have demonstrated that masking both auditory and visual cues reduces the perceived crispiness, with auditory masking having a stronger effect than visual masking (4). These findings indicate that, while multiple sensory modalities contribute to texture perception, auditory cues play a particularly dominant role in judging crispiness. The current result is consistent with previous fMRI findings that listening to food sounds activates the auditory cortex (21). Together with these findings, our results indicate that the crispy sound of low-moisture monaka ice cream stimulates the auditory cortex more effectively than high-moisture ice cream and positively influences the psychological evaluation of food.

In addition, the activity in the vmPFC was higher for low-moisture monaka ice cream than for high-moisture monaka ice cream (Figures 4A, B). Our results are consistent with several neuroimaging findings, demonstrating that the vmPFC is activated during multimodal food processing. Some fMRI studies have reported that the vmPFC shows increased activity when individuals consume or anticipate highly palatable foods, such as sweet or high-fat items, as well as when viewing appetizing food images (9, 10). Further research has indicated that vmPFC activity is not limited to primary sensory pleasure, but also extends to abstract and imagined food rewards, suggesting the integration of multisensory, affective, and cognitive processes (8, 11). The vmPFC is believed to play a central role in encoding the subjective value of food, enabling hedonic comparison and decision-making during food choice. Collectively, these findings establish the vmPFC as a critical hub for integrating sensory pleasure with higher-order reward evaluation mechanisms during food-related experiences.

Furthermore, activity in this region demonstrated a positive correlation with preference ratings (Figures 4C, D). These results are consistent with previous findings that activity in the vmPFC in response to sucrose or fat intake is related to visual stimuli and hedonic ratings, and that the magnitude of this activity correlates with subjective evaluations (12, 22, 23). Moreover, fMRI studies investigating food perception have demonstrated that the vmPFC integrates multisensory information and encodes the hedonic value of food-related experiences. It has been shown that vmPFC activity is modulated not only by gustatory stimuli but also by auditory cues, such as food-related sounds (e.g., crunching or chewing), particularly when these cues enhance subjective pleasure. For instance, studies using simultaneous presentation of taste and sound stimuli have reported that vmPFC activation increases when congruent auditory cues (such as crisp sounds) accompany pleasant tastes, and the degree of vmPFC activation correlates with participants' reported pleasure or palatability (24, 25). Further investigations have also indicated that, when participants listen to appetizing food sounds, vmPFC activation correlates with both the subjective pleasantness of the sound and enhanced hedonic ratings when combined with taste. These findings suggest that the vmPFC serves as a convergence site for auditory and gustatory signals, integrating them into a unified hedonic representation that underpins multisensory flavor and food enjoyment.

Our finding that low-moisture monaka ice cream elicits both positive affect and increased activation in the vmPFC suggests practical implications. Controlling the moisture content of the wafer may enhance not only the perceived taste but also promote positive emotional states, potentially contributing to a modest improvement in wellbeing. It has been reported that low-moisture monaka produces a “crispy” texture and induces pleasurable sensations. However, it remains unclear which sensory modality among the multimodal characteristics of crispiness contributes the most to this pleasant feeling, as well as the corresponding neural activity involved. Future research aimed at generating more pleasant auditory stimuli and analyzing the activity of the auditory cortex in detail can enable the efficient development of monaka ice cream with a higher sensory appeal. Furthermore, conventional assessments of affective responses rely on subjective ratings that are susceptible to bias. Therefore, by employing objective indicators, such as neural activity in the vmPFC, it is possible to develop a monaka ice cream based on unbiased, objective evidence.

Our findings also have theoretical significance in that they suggest the neural basis underlying multimodal food processing. Recent years have seen substantial advances in the fields of neurogastronomy and multimodal food perception, highlighting the complex interplay between sensory modalities in shaping food experiences (26–28). Notably, work by Spence and colleagues has demonstrated that texture, sound, and visual cues can significantly influence both hedonic evaluation and neural responses associated with food enjoyment (29). In line with these insights, our results add to the growing body of evidence that multisensory interactions—particularly the integration of auditory and gustatory signals—play a crucial role in shaping affective food experiences. Furthermore, emerging research has begun to elucidate the neural circuits underlying hedonic assessment of food, implicating brain regions such as the vmPFC, insula, and orbitofrontal cortex in the processing of multisensory food pleasure (27, 30). By situating our findings within this contemporary landscape, we underscore the relevance of neural correlates in understanding the multisensory and hedonic dimensions of food perception. We recognize the need for additional integrative studies that bridge neuroscience, psychology, and sensory science.

### 4.1 Limitations of the study

One important limitation of the present study is the relatively small sample size (*N* = 10), which constrains statistical power and restricts the generalizability of our findings. While our sample size aligns with previous neuroimaging research investigating food-related sensory processing (12), the limited number of participants may hinder the detection of subtle effects and could increase the risk of both Type I and Type II errors. To ensure robust and generalizable conclusions, future studies should aim to replicate and extend our results with larger and more demographically diverse samples.

A second important limitation concerns the timing and context of neural measurements. Specifically, fMRI data were acquired while participants recalled their eating experience through video observation, rather than during the act of consumption itself. This approach likely engages memory-and imagery- related processes; thus, it may not fully reflect neural activity linked to real-time sensory perception of the food. Although efforts have been made in recent studies to capture brain responses during the actual act of eating, real-time neuroimaging during food consumption remains technically challenging. Alternative imaging modalities such as electroencephalography and magnetoencephalography offer superior temporal resolution, but their application during ingestion is hindered by movement artifacts and practical difficulties. Likewise, near-infrared spectroscopy, while less restrictive, can be influenced by artifacts from salivary gland activity during oral processing. Thus, future advances in artifact reduction and imaging technology will be critical for enabling accurate measurement of brain activity in naturalistic eating scenarios. Continued methodological development will deepen our understanding of neural responses during real-time food consumption and help validate findings based on recall or observation paradigms.

Another limitation pertains to the potential for bias arising from the use of video stimuli prepared by Morinaga staff. We acknowledge that reliance on company employees may introduce elements not fully representative of typical consumer behavior, possibly influencing participant perceptions and thus affecting external validity. However, several steps were taken to minimize such bias. The video duration was limited to 16 s and focused exclusively on the mouth and hands of the model, intentionally omitting the eyes or broader facial expressions to avoid nonverbal cues that might suggest differences between monaka samples. Furthermore, both samples were consumed in an equally natural manner, without scripted behaviors or gestures. Notably, the monaka's moisture content—our key variable—was visually imperceptible, ensuring that any cues about crispness could not be inferred from the video's appearance alone. Nonetheless, to further enhance generalisability, future studies should consider employing video recordings featuring a wider range of individuals and consumption styles representative of the broader consumer population.

Finally, the cultural context and generalizability of our findings is also worth considering. Monaka ice cream is a traditional Japanese confectionery that, while increasingly recognized internationally, remains most familiar within Japan and East Asia. Sensory preferences, particularly for texture and sweetness, are known to differ across cultures (31, 32), which may influence both subjective hedonic ratings and associated neural responses to food products. Moreover, the importance attributed to “crispiness” or other texture attributes can vary culturally, potentially modulating consumer expectations and affective experience. Therefore, the current results should be interpreted in light of the participants' cultural background, and caution is warranted in generalizing these findings to populations outside Japan. Future research should investigate cross-cultural differences in the perception and neural representation of multisensory food attributes, to clarify the extent to which these effects apply to diverse consumer populations.

### 4.2 Conclusion

In this study, we used fMRI to measure brain activity immediately after participants consumed monaka ice cream with different moisture contents, while viewing videos designed to evoke recollection of their eating experience. The results showed that the consumption of low-moisture monaka ice cream significantly increased activity in both the auditory cortex and vmPFC. Notably, the crisp “crunch” sound of the low-moisture monaka ice cream elicited responses in the auditory cortex, and activity in the vmPFC was associated with preference ratings. These findings suggest that the multimodal processing of crispy monaka ice cream involves activity in the auditory cortex and vmPFC, the latter of which contributes to the occurrence of subjective pleasurable emotions.

## Data Availability

The original contributions presented in the study are included in the article/Supplementary material, further inquiries can be directed to the corresponding author.
